# Evaluación radiográfica del tercer molar mandibular y su influencia en las patologías del segundo molar mandibular. Estudio transversal

**DOI:** 10.21142/2523-2754-1204-2024-218

**Published:** 2024-11-23

**Authors:** Noelia Ojeda, Yanina Rodríguez, Sol Sanabria, Vicente Fretes, Fátima Bañuelos, José Gamarra

**Affiliations:** 1 Facultad de Odontologia, Universidad Nacional de Asuncion. Asuncion, Paraguay. noelu_253@hotmail.com, yaninajasminr@gmail.com , solsanabria@founa.edu.py , vicentefretes@founa.edu.py , fbanuelos@founa.edu.py , josemgamarra31@gmail.com Universidad Nacional de Asunción Facultad de Odontologia Universidad Nacional de Asuncion Asuncion Paraguay noelu_253@hotmail.com yaninajasminr@gmail.com solsanabria@founa.edu.py vicentefretes@founa.edu.py fbanuelos@founa.edu.py josemgamarra31@gmail.com

**Keywords:** tercer molar, radiografía panorámica, caries dental, reabsorción radicular, third molar, panoramic radiography, dental caries, root resorption

## Abstract

**Objetivo:**

Determinar la posición del tercer molar mandibular y su influencia en la presencia de patologías en el segundo molar mandibular.

**Materiales y métodos:**

Estudio descriptivo, transversal, retrospectivo. Se analizaron 262 radiografías. Por cada radiografía panorámica seleccionada se identificaron el tercer y segundo molar mandibular. Se realizó la clasificación de Pell y Gregory, así como la de Winter. Posteriormente, se evaluó la presencia o ausencia de las siguientes lesiones radiográficas: caries en el segundo molar adyacente, pérdida de hueso periodontal en la cara distal del segundo molar mandibular y reabsorción de la raíz del segundo molar. Los datos de las radiografías fueron almacenados en planillas electrónicas para su tabulación por medio de estadística descriptiva, utilizando el programa informático Excel. Mediante estadística descriptiva se presentan los resultados utilizando tablas con frecuencias y porcentajes. Se empleó la prueba de chi cuadrado para realizar asociaciones de interés mediante el programa R Epi info 7.0.

**Resultados:**

En cuanto a la posición del tercer molar mandibular, la más frecuente fue la IA, y la alveolisis fue la patología más frecuente que afecta al segundo molar mandibular en esta posición. Con relación a la inclinación de las piezas dentarias, la más frecuente fue la mesioangular y la patología más frecuente que afecta al segundo molar en esta inclinación fue también la alveólisis.

**Conclusión:**

La posición IA con inclinación mesioangular del tercer molar mandibular fue la más común, y ocasionó alveolitis en el segundo molar mandibular, la patología más frecuentemente reportada.

## Abstract (en)

**Objective:**

To determine the position of the mandibular third molar and its influence on the presence of pathologies in the mandibular second molar.

**Materials and methods:**

Descriptive, cross-sectional, retrospective study. A total of 262 radiographs were analyzed. For each panoramic radiograph selected, the mandibular third and second molars were identified. The Pell and Gregory classification was performed, as well as Winter's classification, subsequently the presence or absence of the following radiographic lesions was evaluated: caries in the adjacent second molar, periodontal bone loss in the distal aspect of the mandibular second molar, and root resorption of the second molar. The data from the radiographs was stored in electronic spreadsheets for tabulation by means of descriptive statistics using Excel software. Descriptive statistics were used to present the results using tables with frequencies and percentages. The chi-squared test was used to perform associations of interest in means of the R Epi info 7.0 program.

**Results:**

Regarding the position of the mandibular third molar, the most frequent was IA, with alveólisis being the most frequent pathology affecting the mandibular second molar in this position. In relation to the inclination of the teeth, the most frequent was mesioangular and the most frequent pathology affecting the second molar in this inclination was also alveólisis.

**Conclusion:**

The IA position with mesioangular inclination of the mandibular third molar was the most common, causing alveolitis in the mandibular second molar. This is the most frequently reported pathology.

## INTRODUCCIÓN

El tercer molar mandibular es una de las piezas dentarias que, por su posición patológica, podría crear complicaciones tanto locales como regionales ^1^. Eso se debe a que es una de las piezas dentarias más irregulares en cuanto a su morfología y erupción, debido a la falta de espacio entre la cara distal del segundo molar y la rama de la mandíbula ^2^. 

Esto ocasiona que, estas piezas dentarias puedan erupcionar de una manera ectópica o puedan impactarse, lo que se define como una retención de la pieza dentaria por una alteración en su tiempo de erupción. Está definido que los terceros molares mandibulares presentan un mayor porcentaje de impactación en comparación con otras piezas dentarias ^(3, 4)^. 

Debido a su posición y por las alteraciones en su erupción, el tercer molar mandibular es el causante de la afectación patológica en dientes adyacentes, por lo que es el segundo molar mandibular el más afectado ^5^. Entre las patologías más conocidas están la caries en la cara distal, la lisis del hueso o las patologías periodontales como reabsorción radicular ^6^. Existen clasificaciones para la posición e inclinación de los terceros molares, como la de Pell y Gregory, que analiza la posición teniendo en cuenta la relación con la rama de la mandíbula (clase I, II o III) y con el plano oclusal del diente adyacente (posición A, B o C) ^7^. Asimismo, está la clasificación de Winter, que analiza la inclinación del tercer molar y considera el eje longitudinal del tercer molar con relación al eje longitudinal del segundo molar ^8^. 

Existe evidencia de que la impactación o retención de los terceros molares mandibulares se asocian con un mayor riesgo de presentar patologías cercanas o en el segundo molar adyacente ^9^. Es imprescindible un diagnóstico precoz y certero para evitar el avance progresivo de estas patologías, siendo el uso de radiografías panorámicas, una herramienta importante. Este es el primer método que suele ser realizado, ya que tiene la ventaja de proporcionar una amplia perspectiva general de la salud dental y ósea del paciente ^10^. 

Por lo anteriormente mencionado, es recomendable realizar estudios que determinen los patrones de impactación, teniendo en cuenta la posición e inclinación de los terceros molares mandibulares con mayor riesgo a producir patologías ^11^. Por ese motivo, el objetivo de esta investigación fue determinar la relación de la posición del tercer molar mandibular y la presencia de patologías en el segundo molar mandibular. 

## MATERIALES Y MÉTODOS

Se diseñó un estudio descriptivo, transversal y retrospectivo con componente analítico. Se utilizaron radiografías panorámicas registradas en el servicio de Imagenología de la Facultad de Odontología de la Universidad Nacional de Asunción (FOUNA), durante los años 2019 a 2023. El presente trabajo de investigación fue aprobado (P-016-2024) por el Comité de Ética en Investigación (CEI) de la FOUNA. 

La muestra quedó constituida por 262 radiografías, mediante un muestreo no probabilístico. Se incluyeron radiografías panorámicas de pacientes de ambos sexos, mayores de 18 años, de buena calidad imagenológica, con terceros molares con desarrollo radicular completo y que contengan la pieza dentaria adyacente, además que no contengan artefactos por movimiento. Las radiografías que no contaban con estos criterios fueron descartadas. 

**Figura 1:**
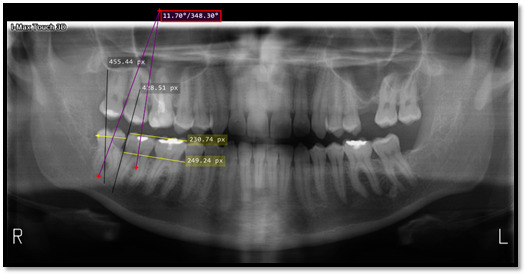
Ubicación de las líneas trazadas para determinar la posición de Pell y Gregory, y Winter.

En primer lugar, en cada radiografía panorámica seleccionada, se identificaron el tercer y segundo molar mandibular. Se trazaron líneas verticales y horizontales para establecer la posición con la clasificación de Pell y Gregory como sigue: dos líneas verticales, una en el borde anterior de la rama mandibular y otra en la cara distal del segundo molar mandibular, y una línea horizontal en sentido mesiodistal, sobre la corona de la pieza dentaria para determinar el espacio disponible (clase I, II o III). Se delimitaron dos líneas horizontales, una en la línea oclusal, establecida por las cúspides de los molares, y otra en la línea cervical, para determinar la profundidad (A, B o C) ^7^. De la misma manera, se delimitó una línea siguiendo el eje longitudinal de la pieza dentaria y de su adyacente para evaluar la relación espacial de la pieza dentaria siguiendo la clasificación de Winter respecto de la inclinación (vertical, horizontal, mesioangular, distoangular, bucoangular, linguoangular o invertida) ^8^ (Fig. 1). 

Posteriormente, se prestó importancia a la presencia o ausencia de las siguientes lesiones radiográficas: caries en el tercer molar y segundo molar adyacente, pérdida de hueso periodontal en la cara distal del segundo molar mandibular y del tercer molar adyacente, y reabsorción de la raíz tanto del segundo como del tercer molar. 

Las imágenes seleccionadas fueron visualizadas y analizadas mediante el programa Micro Dicom Viewer, *software* diseñado para visualizar imágenes médicas en formato DICOM (*Digital Imaging and Communications in Medicine*). El formato DICOM es estándar en la industria médica para el intercambio y almacenamiento de imágenes médicas, como radiografías, tomografías computarizadas (CT), resonancias magnéticas (MRI) y más ^12^. 

Los datos de las radiografías fueron almacenados en planillas electrónicas para su tabulación por medio de estadística descriptiva, utilizando el programa informático Excel. Mediante estadística descriptiva se presentan los resultados utilizando tablas con frecuencias y porcentajes, se utilizó la prueba chi cuadrado para realizar asociaciones entre las variables de interés, considerando la significancia estadística (p < 0,05) y asumiendo un nivel de confianza del 95%, mediante el programa R Epi info 7.0.

## RESULTADOS

Fueron incluidas en esta investigación 262 radiografías de pacientes que acudieron a los servicios clínicos de la Facultad de Odontología de la Universidad Nacional de Asunción entre 2019 y 2023. El 58,4% de las radiografías correspondieron a pacientes del sexo femenino. Con el 47,7 % de las radiografías en rango etario de 20 a 29 años. La edad promedio de los pacientes fue de 32±12 años. 

Más del 80% de los pacientes presentaron patologías en el segundo molar mandibular, se aplicó la prueba de chi-cuadrado para asociar la presencia de patologías en el segundo molar por sexo, y se concluyó que no existe relación estadística entre dichas variables (p > 0,05). 

Sobre la posición e inclinación de los terceros molares mandibulares, la posición IA e inclinación mesioangular fueron las más frecuentes (60,6% y 37,1%, respectivamente), seguidas por la posición IIA y la inclinación vertical (25,1% y 34,6%, respectivamente) (Tabla 1).

**Tabla 1:** Distribución de terceros molares mandibulares según su posición (clasificaciones de Winter y de Pell y Gregory) por lado (n = 431).

Posición de los terceros molares mandibulares		Lado				Total	
		Derecho (n = 220)		Izquierdo (n = 211)		(n=431)	
		Frec.	%	Frec.	%	Frec.	%
Clasificación Winter	Distoangular	3	1,4 %	1	0,5 %	4	0,9 %
	Horizontal	10	4,5 %	7	3,3 %	17	3,9 %
	Mesioangular	128	58,2 %	133	63,0 %	261	60,6 %
	Vertical	79	35,9 %	70	33,2 %	149	34,6 %
Clasificación Pell y Gregory	IA	86	39,1 %	74	35,1 %	160	37,1 %
	IIA	57	25,9 %	51	24,2 %	108	25,1 %
	IIIA	0	0,0 %	5	2,4 %	5	1,2 %
	IB	17	7,7 %	21	10,0 %	38	8,8 %
	IIB	38	17,3%	41	19,4 %	79	18,3 %
	IIIB	5	2,3 %	1	0,5 %	6	1,4 %
	IC	0	0,0 %	2	0,9 %	2	0,5 %
	IIC	5	2,3 %	2	0,9 %	7	1,6 %
	IIIC	12	5,5 %	14	6,6 %	26	6,0 %

Altos porcentajes de segundos molares mandibulares con patologías fueron observados en ambos lados. Los mayores porcentajes de segundos molares mandibulares con patologías se observaron en pacientes cuyos terceros molares mandibulares tenían una posición IA, IIA y IIB. Asimismo, la inclinación mesioangular del tercer molar fue la más prevalente en provocar una patología en su adyacente, seguida de la inclinación vertical. Las patologías del segundo molar mandibular más prevalentes fueron la caries, la reabsorción radicular y la alveólisis, siendo esta última la más frecuente.

En el lado derecho, se identificó a la alveólisis como la patología más frecuente, seguida por la caries dental y por último la reabsorción. La inclinación mesioangular fue la más prevalente con todas las patologías, pero las más frecuentes fueron la alveólisis y la caries. La posición IA está ligada a una mayor frecuencia de alveólisis (Tabla 2).

**Tabla 2:** Distribución de segundos molares inferiores del lado derecho con patologías según tipo de patología y posición de los terceros molares inferiores.

Posición del tercer molar mandibular derecho		Patologías en segundo molar mandibular derecho					
		Caries (n = 85)		Reabsorción (n = 41)		Alveólisis (n = 131)	
		Frec.	%	Frec.	%	Frec.	%
Clasificación Winter	Distoangular	0	0,0%	2	4,9%	2	1,5%
	Horizontal	8	9,4%	6	14,6%	7	5,3%
	Mesioangular	59	69,4%	19	46,3%	83	63,4%
	Vertical	18	21,2%	14	34,2%	39	29,8%
Clasificación Pell y Gregory	IA	18	21,2%	13	31,7%	44	33,6%
	IIA	23	27,1%	8	19,5%	29	22,1%
	IIIA	0	0,0%	0	0,0%	0	0,0%
	IB	10	11,8%	5	12,2%	13	9,9%
	IIB	23	27,1%	9	22,0%	30	22,9%
	IIIB	2	2,4%	1	2,4%	2	1,5%
	IC	0	0,0%	0	0,0%	0	0,0%
	IIC	3	3,5%	2	4,9%	4	3,1%
	IIIC	6	7,1%	3	7,3%	9	6,9%

En el lado izquierdo, al igual que en el lado derecho, se identificó a la alveólisis como la patología más frecuente, seguida por la caries dental y, por último, la reabsorción. También con la inclinación mesioangular, la alveólisis y la caries dental fueron las más frecuentes, y con la posición IA, fueron la alveólisis y la reabsorción. Ahora bien, el más alto porcentaje de caries se observó en pacientes cuyo tercer molar mandibular tenía clase IIB (Tabla 3). 

**Tabla 3:** Distribución de segundos molares mandibulares del lado izquierdo con patologías según tipo de patología y posición de los terceros molares mandibulares.

Posición del tercer molar mandibular izquierdo		Patologías en segundo molar mandibular izquierdo					
		Caries (n = 67)		Reabsorción (n = 52)		Alveólisis (n = 130)	
		Frec.	%.	Frec.	%	Frec.	%
Clasificación Winter	Distoangular	1	1,5%	1	1,9%	1	0,8%
	Horizontal	5	7,5%	3	5,8%	4	3,1%
	Mesioangular	51	76,1%	32	61,5%	94	72,3%
	Vertical	10	14,9%	16	30,8%	31	23,9%
Clasificación Pell y Gregory	IA	14	20,9%	16	30,8%	44	33,9%
	IIA	14	20,9%	16	30,8%	27	20,8%
	IIIA	3	4,5%	1	1,9%	2	1,5%
	IB	3	4,5%	6	11,5%	14	10,8%
	IIB	24	35,8%	9	17,3%	31	23,9%
	IIIB	0	0,0%	1	1,9%	1	0,8%
	IC	1	1,5%	0	0,0%	2	1,5%
	IIC	1	1,5%	0	0,0%	1	0,8%
	IIIC	7	10,5%	3	5,8%	8	6,2%

En resumen, la posición e inclinación más frecuente del tercer molar mandibular fue la IA mesioangular, con una relación significativa (p < 0,002) y con la alveólisis como patología más frecuente en el segundo molar mandibular. Le sigue la posición IIB con la caries dental.

## DISCUSIÓN

El presente estudio determinó la clasificación y posición de los terceros molares mandibulares, y su influencia en la presencia de patologías en el segundo molar mandibular. Se evidenció una relación significativa entre la posición IA, inclinación mesioangular con la presencia de alveólisis en el segundo molar adyacente. 

Al igual que en otros estudios ^(2, 13)^, en este, el mayor porcentaje de radiografías analizadas correspondió al sexo femenino, aunque no se encontró una relación significativa entre la presencia de patologías en el segundo molar mandibular con respecto al sexo. Esto podría deberse a que se relaciona al sexo femenino con un mayor cuidado y conciencia de la salud bucal, lo que aumentaría las visitas al odontólogo en comparación con el sexo masculino ^14^. 

Con respecto a la clasificación de Pell y Gregory, en este estudio se determinó que la clase IA fue la más frecuente, con un 37,1%, dato que coincide con otros estudios relacionados, los cuales determinan que la clase IA es la más frecuente, con porcentajes del 59,8% ^15^ y el 53,5% ^16^. En cuanto a la inclinación, según la clasificación de Winter, la más frecuente fue la mesioangular, en un 60,6%, lo que coincide con otros estudios que determinan que la posición mesioangular es la más frecuente en toda la clasificación, caracterizada por una posición mesial con relación a la ubicación de su adyacente ^17^^-^^19^. 

Sobre las patologías presentes en el segundo molar mandibular con respecto a la posición del tercer molar adyacente, el estudio de Skitiou ^19^ analizó 353 radiografías y determinó que las patologías más frecuentes fueron la alveólisis, la caries dental y la reabsorción radicular, siendo la primera la más frecuente, con una significancia (p < 0,05), al igual que lo reportado en esta investigación. Del mismo modo, un estudio realizado en Brasil en 1192 radiografías determinó que la patología más frecuente fue la alveólisis (50,3%); sin embargo, no encontró una relación significativa entre la posición del tercer molar mandibular y la patología de su adyacente ^20^. Otros hallazgos mencionan que la patología más frecuente es la caries dental ^21^^-^^23^, pero en esta investigación la caries dental se evidenció en un 45,8%. También se evidenció que el 31,7% de los segundos molares presentaron reabsorción radicular similar a otra investigación, con un 23,7% de los casos ^24^. 

Cabe destacar que las diferencias significativas posibles entre un estudio y otro se apegan a las características de cada uno. Esas diferencias podrían deberse, principalmente, al tamaño de la muestra. No obstante, es importante señalar que, cuantitativamente, la alveólisis es la patología más frecuente presentada en el segundo molar mandibular. 

Las piezas dentarias con posiciones IIB e inclinaciones mesioangular vuelven a los segundos molares más propensos a desarrollar caries dental, aunque en este estudio no se encontró una significancia estadística en relación a esta posición y a la patología, podría deberse a qué la pieza dentaria se encuentra parcialmente impactada y recubierta por tejido gingival lo que dificulta la higiene y facilita la acumulación de placa, así como de bacterias ^25^. Teniendo en cuenta la posición, la de mayor frecuencia y que predispone a la presencia de patologías fue la mesioangular, relacionada con un mayor porcentaje al desarrollo de caries y reabsorción radicular, lo que difiere con el trabajo de Arrizumi ^(26^), quien determinó que la reabsorción se relaciona con una posición horizontal, al igual que Ege *et al*. ^27^, los cuales señalan que la posición mesioangular estuvo relacionada con el desarrollo de alveólisis. 

Sobre las limitaciones del trabajo debemos destacar la cantidad de radiografías analizadas, que fueron escasas en comparación con otras investigaciones, así como la calidad de las radiografías y su correcto almacenamiento.

## CONCLUSIÓN

La posición IA e inclinación mesioangular del tercer molar mandibular es la más frecuente en la influencia del desarrollo de alveólisis en el segundo molar mandibular, con una relación significativa. 

## Fuente de financiamiento:

Autofinanciando
